# Nutritional status of a young adult population in saline-prone coastal Bangladesh

**DOI:** 10.3389/fpubh.2023.1095223

**Published:** 2023-06-01

**Authors:** Tapas Mazumder, Shannon Rutherford, Syed Moshfiqur Rahman, Mohammad Radwanur Talukder

**Affiliations:** ^1^Health Research Institute, Faculty of Health, University of Canberra, Canberra, ACT, Australia; ^2^School of Medicine and Dentistry, Griffith University, Gold Coast, QLD, Australia; ^3^Department of Women's and Children's Health, Uppsala University, Uppsala, Sweden; ^4^Maternal and Child Health Division, International Centre for Diarrhoeal Disease Research, Dhaka, Bangladesh; ^5^Wellbeing and Preventable Chronic Diseases (WPCD) Division, Menzies School of Health Research, Darwin, NT, Australia; ^6^College of Health and Human Sciences, Charles Darwin University, Darwin, NT, Australia; ^7^Baker Heart and Diabetes Institute, Human T-cell Leukaemia Virus Type 1 (HTLV-1) Research, Melbourne, VIC, Australia

**Keywords:** nutrition, obesity, salinity, Bangladesh, young adult

## Abstract

**Introduction:**

Like many low- and middle-income countries, understanding the nutritional status of the young population in Bangladesh has had less attention. With projected climate change and associated sea level rise, the existing problem of salinity in coastal Bangladesh will significantly increase and further worsen agrobiodiversity. This research aimed to examine the nutritional status of a young population in climate-vulnerable coastal Bangladesh to inform appropriate intervention strategies to reduce the burden on health and economic outcomes.

**Methods:**

A cross-sectional survey was conducted in 2014, and anthropometric measures were conducted for 309 young people aged 19–25 years in a rural saline-prone subdistrict in southwestern coastal Bangladesh. Body mass index (BMI) was calculated from body height and weight, and data about socio-demographic factors were collected. To identify the socio-demographic risk factors affecting undernutrition (BMI <18.5 kg/m^2^) and overweight/obesity (BMI ≥ 25.0 kg/m^2^), multinomial logistic regression analysis was used.

**Results:**

Overall, one-fourth of the study population was classified as underweight, and nearly one-fifth were overweight or obese. The proportion of underweight was significantly higher in women (32.5%) compared to that of men (15.2%). Overall, employment, especially in women, was associated with reduced odds of being underweight (adjusted odds ratio—aOR: 0.32; 95% confidence interval - CI: 0.11, 0.89). Subjects with secondary education incomplete (grades 6-9) compared to those with primary or below education (grades 0-5; aOR: 2.51; 95% CI: 1.12, 5.59) and employed compared to those unemployed groups (aOR: 5.84; 95% CI: 2.67, 12.74) were more likely to be overweight or obese in this study population. These associations were more pronounced in women.

**Discussion:**

Multisectoral program strategies are required to tackle the growing burden of malnutrition (both under and overweight) in this young age group tailored to local contexts including in climate-vulnerable coastal Bangladesh.

## 1. Introduction

Across the human life-course, nutrition plays a vital role in survival, physical and mental development, productivity, and overall wellbeing (1). Improved nutrition makes the immune system strong, reduces the risk of adverse gestation and childbirth, decreases the likelihood of diabetes and coronary heart diseases, and improves longevity (2–5). Malnutrition among women of reproductive age, such as underweight (body mass index—BMI <18.5 kg/m^2^), is associated with an increased risk of low birthweight, intrauterine growth restriction, neonatal illness and death, and a lack of growth (1, 2). Maternal short stature and underweight status are independent risk factors for preterm birth and small for gestational age in rural Bangladesh (6). Despite this, the double burden of malnutrition remains a major challenge for many parts of the world. Globally, 1.9 billion adults are overweight or obese, while 462 million are underweight, impacting the progress and achievement of several global health and development targets (7, 8). More importantly, over the years, the nutritional status of young people (10–24 years) has been given less attention despite this period being critical for a healthy transition to adulthood and later life (8). In Bangladesh, ~46 million young people (15–29 age group) comprise the working-age group (15–64 years) (9). The health and wellbeing of this group are, therefore, critical to the country's future and economic growth (10). As a key determinant of health and wellbeing, a better understanding of the nutritional status of this young population group is important for program planning and policy formulation so as to avoid potential future burdens on the health system and economy of Bangladesh.

Similar to global trends, Bangladesh has a double burden of malnutrition, including in the young age group. The results of the latest National Demographic Health Survey showed that for young women aged 15–24 years, around 13% were underweight while nearly 17% were overweight and obese (11). Similarly, in young men aged 20–29 years, the proportions of underweight and overweight/obese were around 20% and 13%, respectively (12), indicating the emerging dual problem of undernutrition and overweight. These reported rates of malnutrition also vary across the divisions for both men and women (12). These variations in average rates of malnutrition at the regional or divisional level also suggest further within-division variability in malnutrition rates is likely because of geographical and climatic dissimilarities across Bangladesh (13). Thus, more region-specific research for Bangladesh is important to investigate whether any hotspots for malnutrition can be identified and addressed through appropriately targeted interventions.

Bangladesh is a low-lying country with a 710-km coastline that is vulnerable to sea-level rise (14). The whole coastal zone, including southwestern coastal areas, is heavily affected by saltwater intrusion and increasing salinity (14, 15). According to Bangladesh's Soil Resource Development Institute (SRDI), between 1973 and 2009, soil salinity across the southwest coastal region significantly increased in terms of both overall land areas affected and more areas being exposed to very-high-range salinity levels (15). From 1973 to 2009, with an increasing trend of overall land areas affected by salinity (from 374 to 432 k hectares), almost 60% of this land area is already experiencing salinity at a very high level including salinity values >16,000 μS/cm (15). By 2050, a 39% increase in annual median soil salinity across the coastal zone is projected when all salinity monitoring stations of southwestern districts indicate advancement to high salinity levels experiencing up to >6.00 dS/m (16). This implies a vast majority of land would be unsuitable for all but a few extremely saline-tolerant crops (15), which will have a significant impact on food security in this region.

People living in coastal saline-prone areas are already vulnerable to several health impacts, such as high salt consumption and high blood pressure from a young age due to multiple natural and associated stresses such as sea level rise and elevated water salinity (17, 18). Salinity has affected agrobiodiversity in Bangladesh's coastal regions. A study in a southwestern coastal district revealed that between 1980 and 2018, there was ~15% decrease in agricultural land and an 8% decrease in natural vegetation (19). All the components of household food products, including rice, the staple food of Bangladesh, homestead gardening, and livestock numbers in coastal areas have negatively been affected by salinity (15, 20). Salinity has also caused the extinction of many crops, and as a method of adaptation, farmers now grow high-yielding varieties in place of native crops (21–23). Many farmers do not grow pulses, oilseeds, and other vegetables anymore, focusing instead on shrimp production (23, 24). The change in agrobiodiversity in the coastal region has brought changes in food habits among its residents (25). Several research papers have reported that the change in agrobiodiversity has also hampered food availability, and consequently, the consumption of diverse nutritious foods, such as vegetables, eggs, seasonal fruits, and milk, among the people living in the saline-prone coastal regions has declined (26, 27). Healthy food intake, dietary diversity, and food security are important determinants of nutrition. However, information on the nutritional status of young adult populations in coastal Bangladesh is limited. Therefore, this study aimed to assess the nutritional status of young people aged 19–25 years living in a saline-prone area of southwestern coastal Bangladesh.

## 2. Methods

### 2.1. Study design, setting, and population

We conducted a cross-sectional survey in a rural saline-prone subdistrict, Koyra, of Khulna district in southwestern coastal Bangladesh in May–June 2014. Being part of the exposed coast, this subdistrict is open to the sea, frequently prone to salinity intrusion, and was reported to have the highest salinity concentration in its ground and surface water sources (>2,000 mg/l) in this region, which makes the use of this water unsuitable for domestic and irrigation purposes (28). Detailed study methods were described previously (18). We randomly selected four villages using a probability proportionate sampling (PPS) technique. In the select four villages, the trained research staff listed down all the members aged 19–25 years from each household through household visits. Thus, we have identified a total of 418 young adults aged 19–25 years. Of those, 340 participants were available for interview and health assessment. Of them, 309 participants with successful anthropometric measurements and complete data were included in this analysis (excluding pregnant women *n* = 21, declined to participate *n* = 4, and had inconsistent data *n* = 6).

This study was approved by the Human Research Ethics Committee of Griffith University and Ethical Review Committee of International Center for Diarrhoeal Disease Research, Bangladesh (ICDDR, B). All participants provided written consent at the beginning of data collection.

### 2.2. Outcome measure

There are different methods of measuring nutritional status. Body Mass Index (BMI) is one of the common indicators of nutritional status among adults and used in several previous research studies in Bangladesh (11, 12). The methods of BMI measurement are non-invasive and inexpensive and are easily administered after basic technical training on the standardized procedure (29).

The weight and height of each participant were measured following standard anthropometric techniques. Weight was measured on a digital electric balance (TANITA HD 318 Digital weighing scale, 150 kg ± 0.1 kg), and height was measured using an S þ M height measure scale-2 m (Aaxis Pacific Healthcare, Australia). BMI was calculated using the formula weight (kg)/[height (m)^2^] and categorized into normal weight (18.5–24.9 kg/m^2^), underweight (BMI <18.5 kg/m^2^), and overweight/obese (BMI ≥ 25.0 kg/m^2^) (30).

### 2.3. Covariates

Data on socio-demographic conditions (age, sex, education, occupation, marital status, etc.), household characteristics, the sources of drinking water, and diet of the past 7 days, including consumption of rice, vegetables and fruits, fish, red meat and dairy products, and tobacco use from each of the eligible participants, were collected during household visits. To obtain dietary consumption, we adopted the food frequency questionnaire used in the Bangladesh Integrated Household Survey (31). The questionnaire included 17 food items. For each food item, respondents were asked about the number of days they had consumed each food item in the past 7 days. Education was categorized into primary or below (grades 0–5), secondary incomplete (grades 6–9), and secondary complete or higher (grades 10 or higher). A wealth index score for each household was constructed by applying a principal component analysis (12) of basic housing construction materials (materials used to construct walls, roofs, and floors of houses) and household belongings. The scores were classified into low, middle, and high tertiles.

The salinity of the water samples from drinking water sources identified by the participants was measured in parts per thousand (ppt) using a conductivity meter (Model: Sension5, company: HACH, origin: USA) at the ICDDR, B laboratory. Details about the collection procedure were reported previously (18). Salinity in ppt was converted into milligrams per liter (mg/L; 1 ppt = 1,000 mg/L).

### 2.4. Statistical analysis

Descriptive analyses (frequency distribution, percentage, and mean and standard deviation as appropriate for categorical and continuous variables) were performed to report socio-demographic information of the young adults by their nutritional status (normal weight, underweight, and overweight/obese). Pearson's chi-square test and the *t*-test or ANOVA were conducted as appropriate to estimate and compare the distribution of nutritional status of each explanatory variable. All tests were two-tailed, and a *p*-value of <0.05 was considered statistically significant. To identify the socio-demographic risk factors affecting malnutrition, multinomial logistic regression analysis was used. All the variables from univariate analysis with a *p*-value of ≤ 0.2 were included in the adjusted model that include sex, education, occupation, and wealth index. The estimates of precision were all presented at a 95% CI. STATA version 14.2 was used for data analysis.

## 3. Results

### 3.1. Socio-demographic characteristics

Of the total young adult study population, nearly one-third was classified as underweight, and one-fifth was overweight or obese. Table 1 and Supplementary Table 1 present the socio-demographic characteristics of our study population. The mean height and weight of young adults were 152.4 cm (sd ± 11.5) and 48.9 kg (sd ± 8.4). BMI was not significantly different between men (21.7 ± 4.2 kg/m^2^) and women (20.9 ± 3.9 kg/m^2^; *p* = 0.25). However, men (height 159.7 ± 14.9 cm, range 115.7–179.8 cm; weight 53.9 ± 7.6 kg, range 37.8–92.1 kg) were statistically significantly taller and heavier than women (height 148.7 ± 9.6 cm, range 123.2–174.5 cm; weight 46.0 ± 7.4 kg, range 30.0–74.6 kg; *p* < 0.001). The proportion of underweight was 2-fold higher in women compared to men. However, the proportions of overweight/obese did not differ between the two groups (Table 1). The proportions of underweight and overweight were statistically significantly higher among those with the incomplete secondary education group compared to primary or below groups. The proportion of overweight/obese was almost 4 times higher among the employed group compared to the unemployed groups (*p* < 0.001). Although the proportion of overweight and obesity was higher among high socio-economic groups compared to low and middle socio-economic groups, this difference was not statistically significant. The proportions of malnutrition did not significantly differ by marital status (married vs. not married), drinking water source (surface vs. groundwater), consumption of added salt in meals (yes or no), or household membership number (<5 vs. 5 and above). The mean salinity concentration of drinking water sources in the study area was 877 mg/L (sd ± 518.8), and the maximum salinity concentration was 1,700 mg/L, which was well above the WHO's recommended level (200 mg/L) for palatability of water (33). The salinity concentration of water sources was slightly higher for those who are underweight (891.2 mg/L) and overweight/obese (955.5 mg/L) compared to the normal weight group (847.6 mg/L). But the difference was not statistically significant (Table 1).

**Table 1:** Socio-demographic characteristics of study participants by their nutritional status (*N* = 309).

	**Normal weight**	**Underweight**	**Overweight/ obesity**	***p*-value**
	***n*** **(%)**	***n*** **(%)**	***n*** **(%)**	
Total	174 (56.6)	81 (25.9)	54 (17.5)	
**Characteristics**				
BMI kg/m^2^ [Mean (sd)]	21.0 (1.9)	17.0 (1.4)	27.9 (3.4)	<0.001^c^
**Sex**				
Male	75 (67.0)	17 (15.2)	20 (17.9)	0.003
Female	99 (50.2)	64 (32.5)	34 (17.3)	
**Education**				
Primary or below (grades 0–5)	54 (60.7)	22 (24.7)	13 (14.6)	0.028
Secondary incomplete (grades 6–9)	55 (45.4)	37 (30.6)	29 (24.0)	
Secondary or higher (grades 10 or higher)	65 (65.7)	22 (22.2)	12 (12.1)	
**Marital status**				
Married	110 (55.3)	50 (25.1)	39 (19.6)	0.395
Not married	64 (58.2)	31 (28.2)	15 (13.6)	
**Occupation**				
Unemployed	99 (55.9)	64 (36.2)	14 (7.9)	<0.001
Employed	75 (56.8)	17 (12.9)	40 (30.3)	
**Household size**				
Less than 5	71 (56.8)	31 (23.8)	23 (18.4)	0.873
5 and above	103 (56.0)	50 (27.2)	31 (16.8)	
**Socio-economic status** ^a^				
Low	56 (53.8)	32 (30.8)	16 (15.4)	0.195
Middle	58 (57.4)	29 (28.7)	14 (13.9)	
High	60 (57.7)	20 (19.2)	24 (23.1)	
**Drinking water source**				
Surface water	74 (59.7)	28 (22.6)	21 (17.7)	0.48
Ground water/Tube well	100 (54.0)	53 (28.6)	33 (17.3)	
Drinking water salinity level (mg/L) [Mean (sd)]	847.6 (504.9)	891.2 (513.2)	955 (576.5)	0.401^c^
**Added salt in meals** ^b^				
Yes	83 (53.5)	42 (27.1)	33 (19.3)	0.565
No	91 (59.1)	39 (25.3)	24 (15.6)	

a A wealth index score was constructed for each household using a principal component analysis of basic housing construction materials (materials used to construct walls, roofs, and floors of houses) and household belongings. The scores were divided into low (−1.77, −0.50), middle (−0.48, 0.40), and high (0.41, 2.28) tertiles.

b Adding salt to foods during a meal (does not include salt used during cooking) (32).

c ANOVA test.

### 3.2. Socio-demographic factors associated with malnutrition

Table 2 presents the overall, and Tables 3, 4 present sex-stratified results of multinomial regression analysis examining the association between socio-demographic factors and underweight, and overweight and obesity considering normal body weight as reference.

**Table 2:** Socio-demographic factors associated with malnutrition among the study population using multinomial regression analysis considering normal body weight as reference (*N* = 309).

**Nutritional status**	**Factors**	**Beta**	***p*-value**	**aOR**	**95% Confidence interval aOR**	
					**Lower**	**Upper**
**Underweight**						
	**Sex**					
	Male	Ref				
	Female	0.57	0.113	1.76	0.87	3.56
	**Education**					
	Primary or below	Ref				
	Secondary incomplete	0.29	0.405	1.34	0.67	2.65
	Secondary or higher	−0.31	0.429	0.73	0.34	1.58
	**Occupation**					
	Unemployed	Ref				
	Employed	**−0.88**	**0.016**	**0.42**	**0.20**	**0.85**
	**Socio-economic**					
	**status** ^a^					
	Low	Ref				
	Middle	0.02	0.942	1.02	0.53	1.99
	High	−0.38	0.308	0.68	0.33	1.42
**Overweight/obese**						
	**Sex**					
	Male	Ref				
	Female	**0.91**	**0.015**	**2.50**	**1.19**	**5.24**
	**Education**					
	Primary or below	Ref				
	Secondary incomplete	**0.92**	**0.025**	**2.51**	**1.12**	**5.59**
	Secondary or higher	−0.06	0.894	0.94	0.36	2.43
	**Occupation**					
	Unemployed	Ref				
	Employed	**1.76**	**<0.001**	**5.84**	**2.67**	**12.74**
	**Socio-economic**					
	**status** ^a^					
	Low	Ref				
	Middle	−0.01	0.98	0.99	0.42	2.33
	High	0.63	0.132	1.87	0.83	4.25

a A wealth index score was constructed for each household using a principal component analysis of basic housing construction materials (materials used to construct walls, roofs, and floors of houses) and household belongings. The scores were divided into low (−1.77, −0.50), middle (−0.48, 0.40), and high (0.41, 2.28) tertiles.

The bold values indicate statistically significant.

^b^aOR -Adjusted Odds Ratio.

**Table 3:** Socio-demographic factors associated with malnutrition among the female study population using multinomial regression analysis considering normal body weight as reference (*N* = 197).

**Nutritional status**	**Factors**	**Beta**	***p*-value**	**aOR**	**95% Confidence interval aOR**	
					**Lower**	**Upper**
**Underweight**						
	**Marital status**					
	Not married	Ref				
	Married	**−1.72**	**0.001**	**0.18**	**0.06**	**0.50**
	**Education**					
	Primary or below	Ref				
	Secondary incomplete	0.43	0.315	1.53	0.66	3.54
	Secondary or higher	−0.87	0.126	0.42	0.14	1.27
	**Occupation**					
	Unemployed	Ref				
	Employed	**−1.14**	**0.03**	**0.32**	**0.11**	**0.89**
	**Socio-economic**					
	**status** ^a^					
	Low	Ref				
	Middle	0.03	0.938	1.03	0.47	2.28
	High	−0.62	0.166	0.54	0.22	1.29
**Overweight/obese**						
	**Marital status**					
	Not married	Ref				
	Married	0.03	0.968	1.03	0.21	4.94
	**Education**					
	Primary or below	Ref				
	Secondary incomplete	**1.28**	**0.029**	**3.61**	**1.14**	**11.41**
	Secondary or higher	−0.72	0.328	0.48	0.11	2.07
	**Occupation**					
	Unemployed	Ref				
	Employed	**2.39**	**<0.001**	**10.90**	**4.05**	**29.30**
	**Socio-economic**					
	**status** ^a^					
	Low	Ref				
	Middle	0.04	0.939	1.04	0.33	3.31
	High	0.46	0.408	1.59	0.53	4.79

a A wealth index score was constructed for each household using a principal component analysis of basic housing construction materials (materials used to construct walls, roofs, and floors of houses) and household belongings. The scores were divided into low (−1.77, −0.50), middle (−0.48, 0.40), and high (0.41, 2.28) tertiles.

The bold values indicate statistically significant.

^b^aOR -Adjusted Odds Ratio.

**Table 4:** Socio-demographic factors associated with malnutrition among the male study population using multinomial regression analysis considering normal body weight as reference (*N* = 112).

**Nutritional status**	**Factors**	**Beta**	***p*-value**	**aOR**	**95% Confidence interval aOR**	
					**Lower**	**Upper**
**Underweight**						
	**Marital status**					
	Not married	Ref				
	Married	0.05	0.935	1.05	0.31	3.54
	**Education**					
	Primary or below	Ref				
	Secondary incomplete	0.05	0.941	1.05	0.29	3.81
	Secondary or higher	−1.37	0.135	0.25	0.04	1.53
	**Occupation**					
	Unemployed	Ref				
	Employed	−0.97	0.203	0.38	0.08	1.69
	**Socio-economic**					
	**status** ^a^					
	Low	Ref				
	Middle	0.01	0.983	1.01	0.26	3.98
	High	0.19	0.795	1.21	0.28	5.15
**Overweight/obese**						
	**Marital status**					
	Not married	Ref				
	Married	0.13	0.821	1.14	0.36	3.60
	**Education**					
	Primary or below	Ref				
	Secondary incomplete	0.65	0.332	1.93	0.48	6.81
	Secondary or higher	0.46	0.585	1.58	0.30	8.28
	**Occupation**					
	Unemployed	Ref				
	Employed	0.86	0.258	2.37	0.53	10.62
	**Socio-economic**					
	**status** ^a^					
	Low	Ref				
	Middle	0.01	0.992	1.01	0.24	4.28
	High	0.74	0.311	2.09	0.50	8.70

a A wealth index score was constructed for each household using a principal component analysis of basic housing construction materials (materials used to construct walls, roofs, and floors of houses) and household belongings. The scores were divided into low (−1.77, −0.50), middle (−0.48, 0.40), and high (0.41, 2.28) tertiles. aOR -Adjusted Odds Ratio.

The odds of being underweight decreased by 58% [adjusted odds ratio (aOR) 0.42; 95% CI 0.20, 0.85] among those employed compared to the unemployed groups. This effect was more pronounced among employed women (aOR: 0.32; 95% CI 0.11, 0.89; Table 3). Overall, the odds of being overweight and obese were 2.5 times higher in women compared to men (aOR: 2.50; 95% CI 1.19, 5.24), 2.5 times higher among those reporting incomplete secondary education compared to the primary or below group (aOR: 2.51; 95% CI 1.12, 5.59), and almost 6 times higher among the employed group compared to the unemployed group (aOR: 5.84; 95% CI 2.67, 12.74) in the adjusted model (Table 2). Education (secondary incomplete) and employment remained statistically significant for overweight/obesity in women in sex-stratified analysis (Table 3). No statistically significant socio-demographic factors associated with malnutrition in young men were observed (Table 4).

### 3.3. Frequency of consumption of different food groups

**Figure 1:**
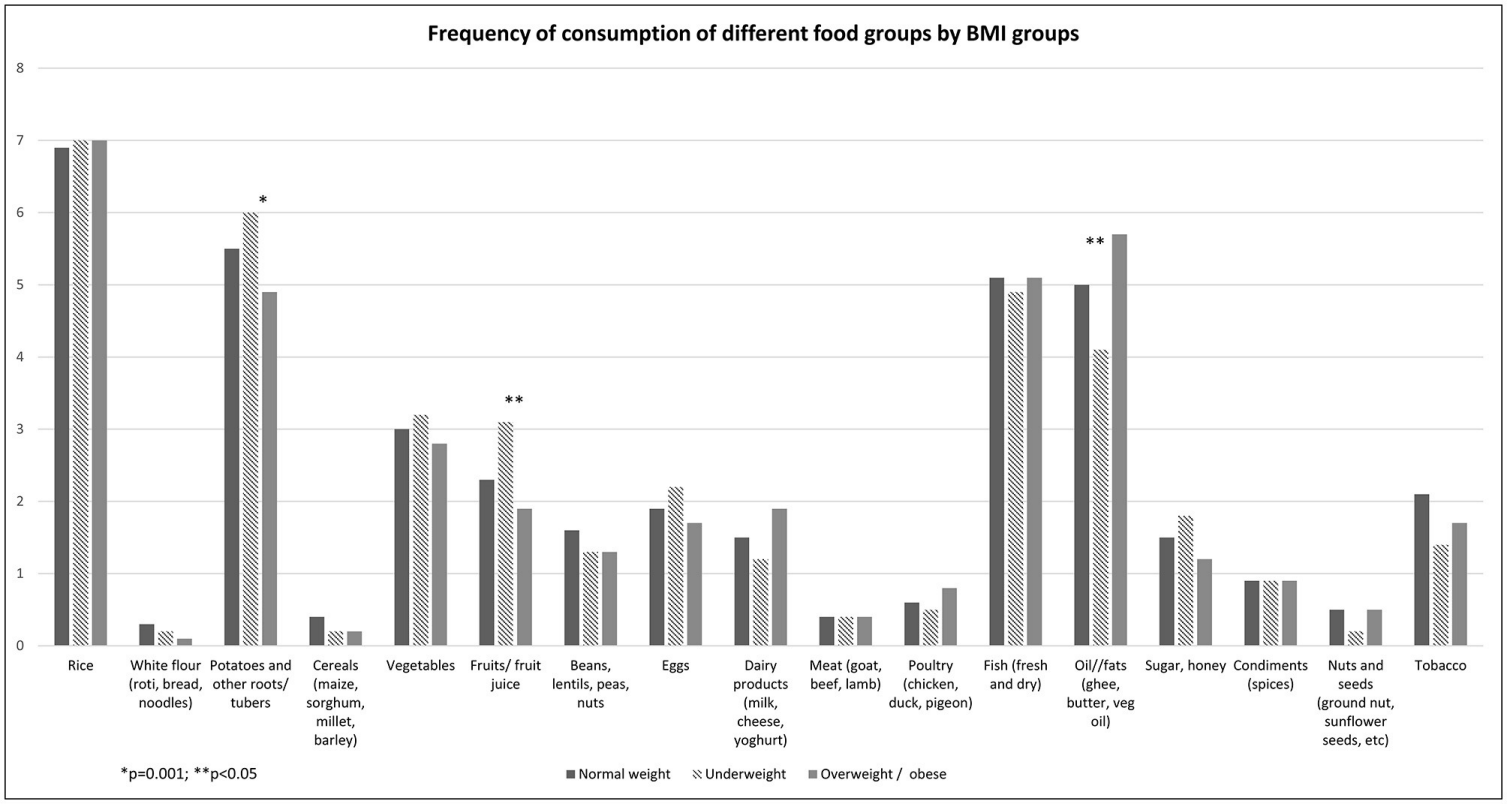
Frequency of comsumption of different food groups by BMI groups.

Figure 1 shows the frequency of consumption of different food groups in past 7 days by nutritional status of study population. Rice was consumed 7 days a week by each nutritional group. Potatoes were consumed 6 days a week by the underweight group and <5 days a week by the overweight/obese group, and this difference in potato consumption was statistically significant (*p* = 0.0014) between groups (Figure 1). On average, fish was consumed 5 days a week and did not differ by nutritional status. Oil and fat consumption was significantly higher in the overweight and obese groups and lower in the underweight group (*p* = 0.015). Consumption of vegetables was, on average, 3 days a week and did not differ by nutritional status. Although fruit consumption was not common, the underweight group had significantly more days of consumption compared to the other two groups (*p* = 0.013). Protein-rich food (poultry, meat, eggs, dairy, beans, lentils, etc.) (Figure 1) appears to be infrequently consumed across each nutritional group. Cereals (maize, barley, millet, etc.) are rarely consumed by any of the nutritional groups.

## 4. Discussion

Our study provides insight into the nutritional status of the critical young population group aged 19–25 years in climate-vulnerable coastal Bangladesh (34). The nutritional status of this young age group in this setting and across Bangladesh is scarce. Both underweight and overweight/obese were observed with high proportions in our young adult study population, disproportionately affecting women. The proportion of underweight among young adult women in our study was considerably higher (32%) than that reported among the 20–29-year age group in BDHS surveys (19.5% in 2014) and that of females of reproductive age (15–49 years) of the Khulna division (13.7% in 2014) (11, 35, 36). However, the proportion of underweight in our young male study population was lower than that of nationally representative young adult men of the 20–29 age group (29.4% in 2011 and 20.4% in 2017–2018) (11, 35). Nearly one in five young adults in our study population were classified as overweight/obese. Women were 2.5 times more likely to be overweight than men. Figures from the BDHS 2011 and 2017–2018 surveys also revealed a similar picture for a comparative age group at the national and regional (Khulna) levels (11, 35). The prevalence of overweight among females in India has also been remarkably higher than that of Indian males as per the National Family Health Surveys (30). According to the literature, changes in occupation patterns and subsequent decreases in physical activity, particularly among women, contribute to more overweight and obesity compared to men (37). Moreover, women often are being more sedentary than men and appear more vulnerable to the effects of energy-dense foods on excess weight gain. Sociocultural beliefs and values around physical activity and fatness also appear to have a greater effect on gender disparities in overweight and obesity (37). An in-depth exploration of factors, such as physical activity, dietary patterns, and beliefs on body image by gender in a sociocultural context, will be critical to designing and targeting appropriate and effective interventions.

The fact that half of the female young adults, also of reproductive age, in our study were either underweight or overweight is of concern. These two forms of malnutrition pose significant health risks to women's reproductive health and child health outcomes. Being overweight during pregnancy may lead to gestational diabetes mellitus (GDM), the baby being large for gestation age, high birthweight of the baby, macrosomia, and cesarean section (38–40). There is also evidence regarding the association between being overweight and preeclampsia (41). On the other hand, being underweight pre-pregnancy is a risk factor related to small for gestational age and low-birthweight babies (39). Impacts of both under and overnutrition at an early age have long-term consequences on health outcomes in both males and females, which in turn lead to poor productivity, therefore further compromising food security and consumption (42, 43).

Our findings suggest that employed participants, especially women, were less likely to be underweight but more likely to be overweight compared to the unemployed group. This is probably because of food affordability relative to income that drives consumption. A similar observation was found in a study in India that monthly income was associated with overweight and obesity in Indian adults (44). While employment gives access to and autonomy of regular income to purchase different commodities, including food, young people are also susceptible to unhealthy food choices (45). The readily available readymade foods and highly sweetened beverages are more convenient and highly desirable, especially to young people. These are high in calories and fat content, making young people susceptible to an obesogenic environment and unhealthy eating habits (45). Moreover, the contribution of young people's income to the overall household economy and competing priorities of household expenditure over food may result in compromising the quality of their food intake (46). Financial constraints can lead to the consumption of cheap, high-energy staple foods, primarily carbohydrates and fats, rather than nutritionally dense food (46, 47). The Cochrane Systematic Review of 1 randomized controlled trial (RCT), 10 cluster RCTs, and 14 prospective controlled studies of income-generating interventions and interventions targeting food prices showed very low evidence that these interventions led to improved expenditure on healthy foods (48). A similar observation was also made by Akter et al. (49) in a national cross-sectional survey in Bangladesh that demonstrated more than half of Bangladeshi adolescent girls and boys consumed an inadequately diversified diet with a higher prevalence of inadequate dietary diversity among girls (49). In this study, lower education levels, household food insecurity, and lower socio-economic conditions were associated with increased chances of having inadequate dietary diversity in both sexes, although it did not report young people's employment status (49). Therefore, further research is recommended to explore whether the employment and income of the young population influence their healthy food choice and consumption.

In this research, the consumption of vegetables, beans, lentils, peas, and nuts was remarkably low among all the nutritional groups. This could be due to the change in local availability of these food items due to changes in crop production and large-scale agribusiness investments associated with salinity changing the agricultural landscape and farming practices (21–24). Projected climate change and sea level rise also indicate a looming stress on crops and livestock production because of increasing and expanding salinity in the coastal belt of Bangladesh (15, 16). Low consumption of dairy, poultry, and meat by the study participants is also reflected in our research. The number of livestock in the saline-prone coastal area has been decreasing due to the reduction of natural vegetation and agricultural land (15, 20). By 2050, annual median soil salinity is projected to increase by 39% across the coastal zone (16). This implies only very few extremely saline-tolerant crops will be available to grow, which will have a significant impact on food security and dietary diversity in this region (15, 26). This may also lead to micronutrient deficiency in the long run and have adverse consequences on maternal and child health (50, 51). Also, noteworthy is the compounding risk of high salt exposure on adverse maternal and child health outcomes in this setting (52). This implies the promotion of healthy food consumption from early life is essential for the vulnerable coastal population.

Our study shows that the nutritional status of the men who participated in our study was not very different from the nutritional status of males at the national level. Rather the proportion of underweight was even lower than that of the males at the national level. However, the higher proportion of overweight or obese among the young men in our rural study population is comparable to national and regional prevalence (12). Although we were unable to determine significant risk factors associated with malnutrition in young men in our study, because of increasing trends of overweight/obesity, it is important to consider further exploratory study in this population group to understand their food consumption behavior for adopting appropriate intervention strategies. In contrast, the nutritional status of women at our study site was very different from the nutritional status of the females at the national level. The higher proportion of underweight among young females is a cause for concern given the long-term consequences of undernutrition on maternal and child health, suggesting investment in targeted nutrition interventions. Although this study did not collect data on micronutrient status, the low consumption of vegetables, beans, lentils, peas, and nuts indicates the risks of “hidden hunger,” that is, deficiencies of essential vitamins and minerals (53). The health and wellbeing of the young population must be prioritized to achieve and sustain Bangladesh's development goals considering this population's significant contribution to the country's economy.

Addressing malnutrition in this region including in the young population will require the involvement of multiple relevant sectors to design and implement appropriate interventions targeting food availability, affordability, and access. To date, several interventions, such as cropland elevation, restriction of saltwater aquaculture, and improved irrigation have been tested; however, limited initiatives and support from NGOs and the government have been some of the key challenges for the sustainability of these interventions (15, 54). The government has also taken strategies to promote and adopt several interventions via its climate action plan, for example, floating gardens, seaweed cultivation, vertical farming using rainwater, a combination of floating farms and fish farms toward safeguarding food security in this region (55). Interventions should also consider the establishment of an alternative food supply chain for the salinity-affected areas to address food availability and access and reduce the potential for elevated salt consumption in this region.

The findings of this research should be interpreted in the context of several limitations. Participation of more women in the survey was observed because of the daytime administration of the survey and the limited opportunity to follow up with the absent participants. This could overestimate the high proportion of overweight/obese and underweight in the female population. However, the study findings are still significant in the context of demonstrating the presence of a double burden of malnutrition in a neglected population group from a geographically vulnerable area of Bangladesh. Also, some important behavioral risk factors, such as physical activity, were not considered in the study. Food consumption pattern was only limited to the frequency of consumption, which is likely to be subject to reporting bias, and actual consumption of different food categories was not available in this research. Finally, as this was a cross-sectional study, we were not able to establish the temporal relationship between the outcome and explanatory variables.

## 5. Conclusion

The high proportion of malnutrition among a young population in a climate-vulnerable rural coastal area of Bangladesh warrants the targeted design of a multi-sectoral program approach to preventing the economic and health consequences associated with poor nutrition. The forecasted effects of climate change, including rising sea levels and salt exposure, indicate that there will be persistent food diversity and supply challenges in low-lying coastal regions. As a result, there is a need for greater emphasis on agro-diversity and alternative food supply chains for the coastal population.

## Data availability statement

The raw data supporting the conclusions of this article will be made available by the authors, without undue reservation.

## Ethics statement

This study was approved by the Human Research Ethics Committee of Griffith University and Ethical Review Committee of ICDDR, B. All participants provided their written informed consent prior to participate in this study.

## Author contributions

TM and MT conceptualized the idea of the manuscript. TM developed the first draft of the manuscript, addressed the comments of the coauthors, and worked on the revised versions as the first author. MT analyzed the data, assisted in writing the background, methods, results, and discussion sections. SMR and SR advised on the data analysis and presentation of results, reviewed the manuscript, and provided feedback to improve its technical rigor. MT and SMR provided overall guidance in the development of the manuscript as senior authors. All authors contributed to the article and approved the submitted version.
