# Supplementary material for: Nutritional status of a young adult population in saline-prone coastal Bangladesh
Source: Front Public Health. 2023 Jun 1;11:1095223. doi: 10.3389/fpubh.2023.1095223 (PMC10267342; doi:10.3389/fpubh.2023.1095223)
Supplement: Supplementary file 1 [file Table_1.docx]

Supplementary Table 1 Body mass index of study participants by their nutritional status and sociodemographic characterisitics (n=309)

|  | **Normal weight** | **Underweight** | **Overweight/ obesity** |
| --- | --- | --- | --- |
| N (%) | 174 (56.6) | 81 (25.9) | 54 (17.5) |
| BMI kg/m^2^ [Mean (sd)] | 21.0 (1.9) | 17.0 (1.4) | 27.9 (3.4) |
| **Sex** [Mean (sd)] |  |  |  |
| Male | 20.7 (1.8) | 16.6 (2.2) | 28.6 (4.2) |
| Female | 21.2 (1.9) | 17.1 (1.0) | 27.5 (2.9) |
| **Education** [Mean (sd)] |  |  |  |
| Primary or below (grades 0-5) | 20.9 (2.0) | 17.1 (1.2) | 29.3 (4.4) |
| Secondary incomplete (grades 6-9) | 21.1 (1.8) | 17.0 (1.6) | 27.9 (3.4) |
| Secondary or higher (grades 10 or higher) | 21.0 (1.8) | 17.1 (1.1) | 26.3 (1.1) |
| **Marital status** [Mean (sd)] |  |  |  |
| Married | 21.1 (1.9) | 17.1 (1.4) | 27.7 (3.1) |
| Not married | 20.8 (1.8) | 17.0 (1.2) | 28.5 (4.2) |
| **Occupation** [Mean (sd)] |  |  |  |
| Unemployed | 20.8 (1.8) | 17.0 (1.1) | 26.9 (2.1) |
| Employed | 21.3 (1.9) | 17.1 (2.1) | 28.2 (3.8) |
| **Household size** [Mean (sd)] |  |  |  |
| Less than 5 | 21.3 (2.0) | 17.2 (0.9) | 28.0 (3.4) |
| 5 and above | 20.8 (1.7) | 16.9 (1.6) | 27.8 (3.5) |
| **Socioeconomic status^a^** [Mean (sd)] |  |  |  |
| Low | 20.9 (2.0) | 16.8 (1.7) | 27.7 (2.8) |
| Middle | 21.0 (1.7) | 17.1 (1.1) | 28.5 (3.9) |
| High | 21.1 (1.9) | 17.3 (1.1) | 27.7 (3.7) |
| **Drinking water source** [Mean (sd)] |  |  |  |
| Surface water | 21.2 (1.8) | 17.1 (1.7) | 28.6 (3.6) |
| Ground water/ Tube well | 20.9 (1.9) | 17.0 (1.2) | 27.4 (3.3) |
| **Added salt in meals^b^** [Mean (sd)] |  |  |  |
| Yes | 21.2 (2.1) | 16.8 (1.7) | 28.4 (3.7) |
| No | 20.9 (1.6) | 17.3 (0.9) | 27.3 (3.1) |

^a^ A wealth index score was constructed for each household using a principal component analysis of basic housing construction materials (materials used to construct walls, roofs, and floors of houses) and household belongings. The scores were divided into low (-1.77, -0.50), middle (-0.48, 0.40) and high (0.41, 2.28) tertiles.

^b^ adding salt to foods during a meal (does not include salt used during cooking)
